# Effects of Source/Drain Electrodes on the Performance of InSnO Thin-Film Transistors

**DOI:** 10.3390/mi13111896

**Published:** 2022-11-02

**Authors:** Qi Li, Dedong Han, Junchen Dong, Dengqin Xu, Yue Li, Yi Wang, Xing Zhang

**Affiliations:** 1 School of Integrated Circuit, Peking University, Beijing 100871, China; 2 School of Information & Communication Engineering, Beijing Information Science and Technology University, Beijing 100101, China; 3 Key Lab of Integrated Microsystems, Peking University Shenzhen Graduate School, Shenzhen 518055, China

**Keywords:** InSnO (ITO), thin-film transistors (TFTs), contact resistance, source/drain electrodes, bias stress stability

## Abstract

Oxide thin-film transistors (TFTs) are of increasing interest in the field of advanced displays. In this work, we explore Al, InSnO (ITO), Ti, and Mo as source/drain electrodes of ITO TFTs. A comparison study is conducted on the electrical properties of ITO TFTs with the four categories of source/drain electrodes. Interestingly, the ITO TFT with an Al source/drain electrode exhibits better device performance, such as a field-effect mobility (μ_FE_) of 26.45 cm^2^/Vs, a reasonable turn-on voltage (V_ON_) of 2.7 V, and a steep subthreshold swing (SS) of 201.50 mV/decade. The contact properties of ITO TFTs are further analyzed, and the results show that the device with an Al electrode exhibits lower contact resistance than the other devices. However, the devices with the four electrode materials all reveal excellent stability under negative bias illumination stress (NBIS) with |ΔV_TH_| < 1 V. This work paves the way for the practical applications of ITO TFTs in next-generation displays.

## 1. Introduction 

Oxide thin-film transistors (TFTs) are promising candidates for next-generation transparent and flexible displays due to their high transmittance, high mobility, low cost, and low temperature process [1,2,3,4,5]. Among all kinds of oxide semiconductor materials, InGaZnO (IGZO) is the most represented category and has been studied extensively in recent years [6,7,8,9,10]. However, the typical mobility of IGZO TFTs is only around 10 cm^2^/Vs, which is not high enough to satisfy the increasing demands of next-generation displays with ultra-fast frame rates and ultra-high resolutions. Therefore, other novel oxide materials, such as InSnO (ITO), InZnO (IZO), ZnON, AlZnO (AZO), and ZnSnO (ZTO), that exhibit high carrier mobility have been extensively investigated recently to replace IGZO [11,12,13,14,15]. 

It is known that the contact resistance between the channel layer and source/drain electrode is a bottleneck factor on the performance of oxide TFTs, which is responsible for limiting the transconductance (hence the device mobility) [16,17,18,19]. However, as the display size and resolution increase, the reduction of RC load of display is in urgent need, which also requires low-resistance metals for the source/drain electrodes of oxide TFTs [20]. Therefore, exploration of suitable source/drain electrode materials is necessary to realize the practical applications of oxide TFTs in large-area and advanced displays. Much research has been carried out regarding low-resistance source/drain electrodes on the performance of oxide TFTs [21,22,23,24,25,26]. According to previous reports, conducting materials such as Al, ITO, Ti, and Mo are commonly used as source/drain electrodes since they are more likely to form ohmic contacts, which is beneficial to achieve high-performance oxide TFTs.

ITO is well-known as a transparent conductive material and has been widely used in display applications for transparent electrodes. Recently, utilization of ITO as a channel material has been a research hotspot in the field of oxide TFTs due to its great potential to achieve high mobility [11,27,28]. In our previous work, ITO was investigated as a channel layer of TFTs, and excellent electrical characteristics were obtained [29]. However, our previous studies did not investigate source/drain electrode materials of ITO TFTs. Therefore, in this work we investigate source/drain electrode materials, exploring Al, ITO, Ti, and Mo as source/drain electrodes. The results demonstrate that the device with an Al source/drain electrode exhibits the best performance with a high field-effect mobility (μ_FE_) of 26.45 cm^2^/Vs, a reasonable turn-on voltage (V_ON_) of 2.7 V, and a steep subthreshold swing (SS) of 201.50 mV/decade. We hope that our work can accelerate the practical applications of ITO TFTs in next-generation displays.

## 2. Experimental Methods

**Figure 1:**
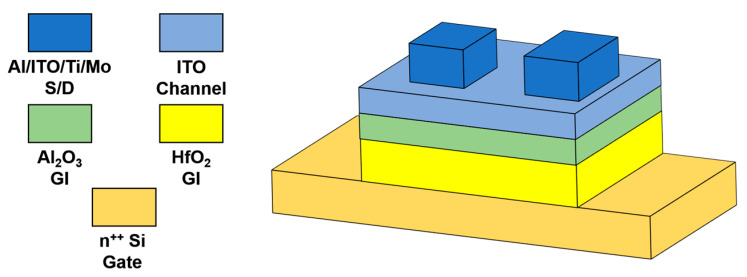
Schematic device structure of ITO TFTs.

As shown in Figure 1, bottom-gate top-contact ITO TFTs were fabricated in this work. A heavily-doped n-type Si wafer was used as both the substrate and gate electrode. Firstly, the Si wafer was ultrasonic cleaned in acetone, alcohol, and deionized water for five minutes, respectively. Subsequently, a bi-layer gate insulator (HfO_2_/Al_2_O_3_) was prepared. 30 nm thick HfO_2_ was deposited using a radio-frequency (RF) magnetron sputter (Beijing Jinshengweina, JR-2B) in an Ar/O_2_ = 90/10 atmosphere at room temperature, and 10 nm thick Al_2_O_3_ was deposited using an ALD system (MEZ, M-150) at 100 °C. The precursors used for deposition of Al_2_O_3_ were trimethylaluminum and deionized water. Afterwards, 5 nm thick ITO channel layer was sputtered in an Ar/O_2_ atmosphere with an 80/20 ratio at room temperature. Finally, four kinds of source/drain electrodes were sputtered at room temperature, including Al, ITO, Ti, and Mo. The devices were patterned using conventional photolithography (SUSS, MJB4) and a lift-off process.

Channel dimensions of the ITO TFTs were 100 μm × 100 μm. The size of the S/D electrode was 60 μm × 100 μm. Thermal annealing was performed after source/drain electrode deposition under vacuum at 100 °C for 1 h. The electrical characteristics were evaluated using an Agilent B1500A semiconductor device analyzer. The interface properties of the S/D electrode and ITO channel were examined using transmission electron microscopy (TEM, FEI Tecnai F20).

## 3. Results and Discussion

**Figure 2:**
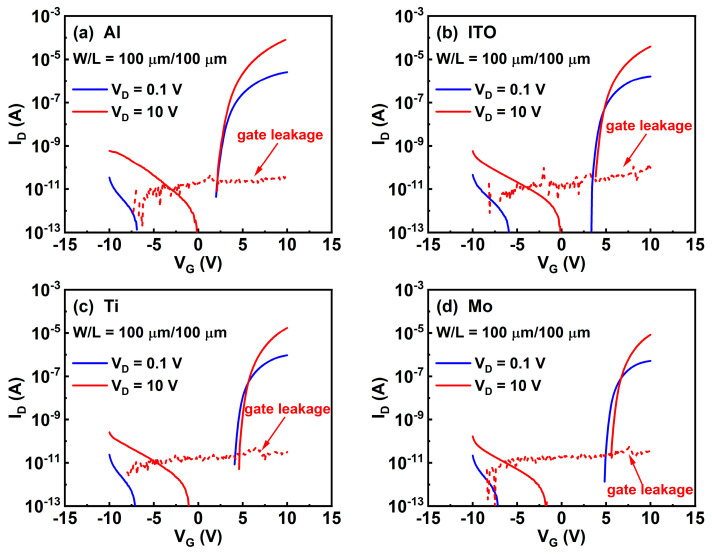
Transfer curves of annealed ITO TFTs with (**a**) Al, (**b**) ITO, (**c**) Ti, and (**d**) Mo source/drain electrodes.

To determine the effects of the electrode materials on the electrical properties of the ITO TFTs, typical transfer curves were firstly measured, as shown in Figure 2a–d. The gate bias was applied from −10 to 10 V with drain voltages of 0.1 and 10 V. The gate leakage current (I_GS_) is also provided in Figure 2. For all devices, I_GS_ is lower than 100 pA, which is negligible compared to I_D_. To analyze the samples quantitively, we extracted some important parameters, as listed in Table 1. The μ_FE_ is the linear mobility and is determined by the maximum transconductance (g_m_) at a drain voltage of 0.1 V. It is calculated using Equations (1) and (2).

g_m_ = ∂I_D_/∂V_G_ (1)

μ_FE_ = g_m_L/WC_OX_V_D_ (2)

C_OX_ is the gate insulator capacitance per unit area, L is the channel length, and W is the channel width [30]. V_ON_ is defined as the gate voltage that induced a drain current of 1 nA. SS is estimated using Equation (3) [31].

SS = [∂log(I_D_)/∂V_G_]^−1^ (3)

**Figure 3:**
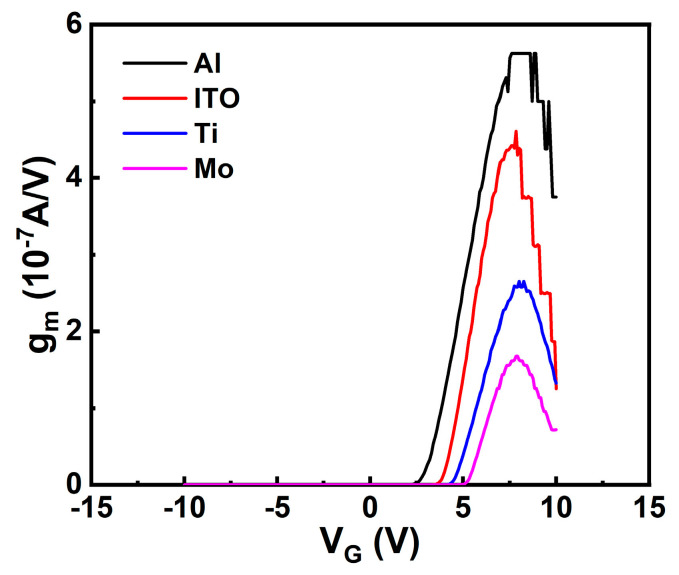
g_m_-V_G_ plots measured for annealed TFTs with Al, ITO, Ti, and Mo S/D electrodes.

Notably, the device with an Al source/drain electrode after annealing revealed better electrical properties than the other devices, showing a high µ_FE_ of 26.45 cm^2^/Vs, a reasonable V_ON_ of 2.7 V, and a steep SS of 201.50 mV/decade. The excellent electrical performance of the ITO TFT with an Al source/drain electrode is comparable to our previous work [29], which demonstrates the feasibility of ITO materials for TFTs. The above results illustrate that Al is a suitable electrode material for ITO TFTs.

g_m_ as a function of V_G_ was also extracted and is shown in Figure 3. The four devices reach a maximum g_m_ (hence μ_FE_) at a close V_G_, while the device with an Al electrode exhibits a larger maximum g_m_ (hence μ_FE_) than the other devices. The presence of a maximum g_m_ (hence μ_FE_) can be attributed to the material properties of the channel layer. Since ITO is amorphous, the trap density is low and a large μ_FE_ can be achieved when the small potential barriers associated with structural disorder are surpassed, where μ_FE_ is maximum. As V_G_ increases, the conductive channel is drawn closer to the ITO/Al_2_O_3_ interface, which contributes to increased scattering effects resulting in a decrease of μ_FE_ [1].

**Figure 4:**
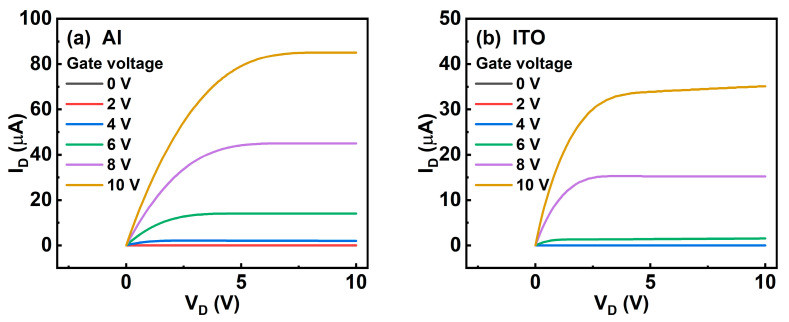

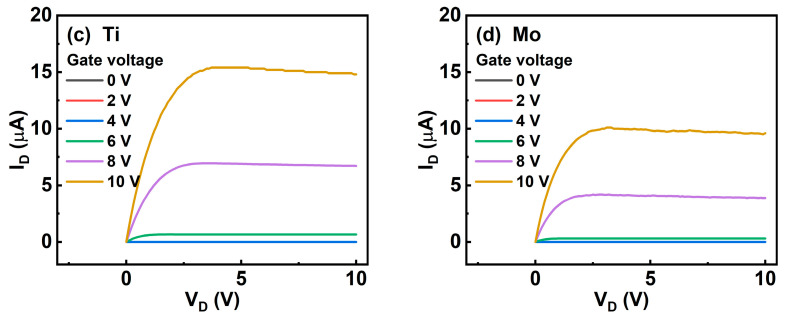
Output curves of annealed ITO TFTs with (**a**) Al, (**b**) ITO, (**c**) Ti, and (**d**) Mo source/drain electrodes.

Figure 4 shows the output curves of annealed ITO TFTs with the four different source/drain electrode materials. The device with an Al electrode (Figure 4a) exhibits the largest output current with a distinct linear and saturation region, which is consistent with transfer curves. The device with an ITO electrode (Figure 4b) exhibits a lower output current than the device with an Al electrode. For the devices with Ti and Mo electrodes (Figure 4c,d), the saturation current presents a trend that increases first and then decreases with drain voltage, which may be caused by the large vacancies, traps, and interface charges between the channel layer and the source/drain electrodes. In addition, there is a jitter of the saturation current in Figure 4c,d. Therefore, the output characteristics also demonstrate that Al is a suitable electrode material for ITO TFTs.

**Figure 5:**
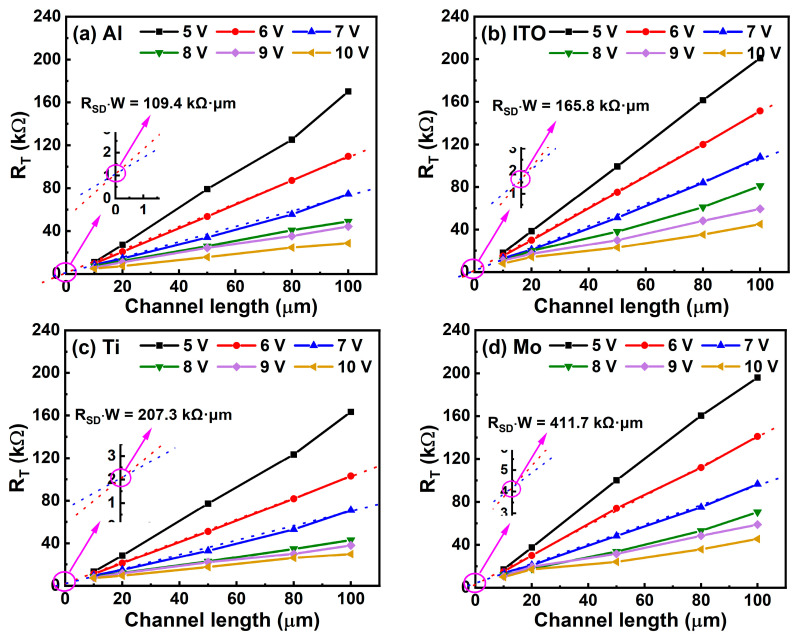
R_T_ of ITO TFTs with (**a**) Al, (**b**) ITO, (**c**) Ti, and (**d**) Mo source/drain electrodes as a function of channel length with different V_G_. The insets show intercepts by zooming in on R_T_-axis.

Here, r_ch_ is the channel resistance per unit channel length. Figure 5 shows the R_T_ as a function of the channel length (L) at various gate voltages for the ITO TFTs with different source/drain electrode materials. Considering that the lines related to V_G_ of 6 V and 7 V in all Figures (Figure 5a–d) show better linearity, we used those two lines for linear fitting to obtain the value of R_SD_, as shown in the insets in Figure 5a–d. After applying linear fitting, the width-normalized contact resistance (R_SD_ × W) of the TFTs with Al, ITO, Ti, and Mo electrodes were calculated to be 109.4, 165.8, 207.3, and 411.7 kΩ·μm, respectively. Of note, the R_SD_ presents transfer and output curves with a consistent trend. The contact property indicates that ideal ohmic contact can be readily formed between the ITO channel layer and Al electrode. However, a comparison with recent reports of oxide TFTs with different source/drain electrodes is exhibited in Table 2. Our ITO TFT with an Al source/drain electrode shows competitive characteristics.

Previous studies have reported that the contact resistance of the source/drain electrode and channel layer decreases with the decreasing work function of the source/drain electrode. The electrode with the lower work function is more likely to form ohmic contact. It was previously reported that the work functions of Al, ITO, Ti, and Mo are 4.28 eV, 4.32 eV, 4.33 eV, and 4.60 eV, respectively [32,33]. Therefore, the ITO TFT with an Al source/drain electrode shows the best performance possibly due to its low work function.

For further evaluating the contact property between the ITO channel layer and different source/drain electrode materials, the transmission line method (TLM) was utilized to extract contact resistance (R_SD_) [34]. The total resistance (R_T_) was defined as the sum of channel resistance (R_ch_) and R_SD_ at V_D_ = 0.1 V by the following equation:

R_T_ = V_D_/I_D_ = R_ch_ + R_SD_ = r_ch_L + R_SD_ (4)

**Figure 6:**
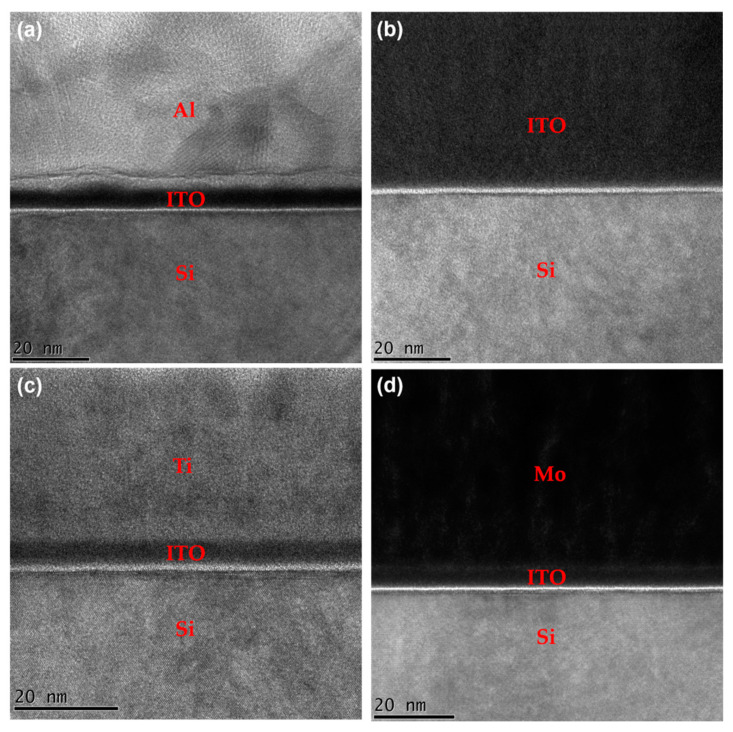
TEM images of the interfacial microstructures between ITO and the metal electrodes: (**a**) Al, (**b**) ITO, (**c**) Ti, and (**d**) Mo.

To achieve a more in-depth study on various metal electrodes and their effects on electrical performance, TEM measurements of the electrodes/ITO interface were performed, as shown in Figure 6a–d. All samples were annealed under vacuum at 100 °C for 1 h, which is the same annealing condition for the devices. There was no evidence of an interfacial reaction between the electrodes and ITO for ITO, Ti, and Mo even though the samples were annealed after metal deposition. However, an aluminum oxide (AlO_x_) layer was formed at the interface between the ITO and Al. As shown in Table 1 and Figure 5, the TFT with an Al electrode exhibits better electrical properties and lower R_SD_. This can be attributed to the electron transfer from AlO_x_ to ITO that would occur at the initial interface since the electronegativities of In and Sn are slightly larger than that of Al [23,42]. This induces an increase in carrier density on the ITO surface, leading to the reduced R_SD_ between the Al and ITO.

**Figure 7:**
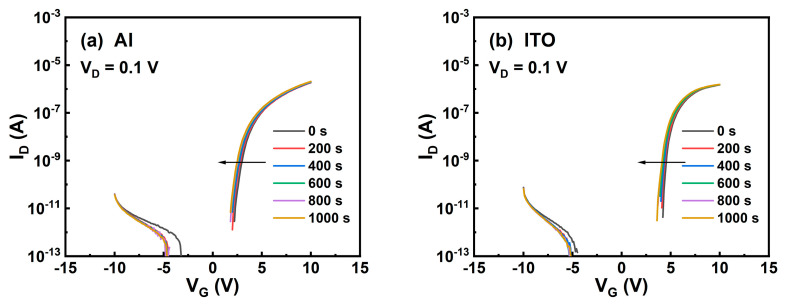

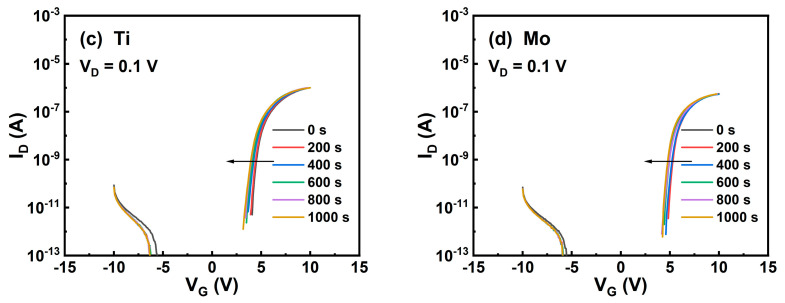
NBIS time-evolutions of the transfer curves for the ITO TFTs with (**a**) Al, (**b**) ITO, (**c**) Ti, and (**d**) Mo source/drain electrodes.

As TFTs are almost inevitably exposed to visible light originating from the underlying backlight unit in a working AMLCD panel or the self-emitting radiation in AMOLED products, the stability of the TFTs with the four categories of source/drain electrodes under negative bias illumination stress (NBIS) was measured, as shown in Figure 7. For NBIS tests, the devices were stressed under a negative gate bias (V_GS_ = −4 V, V_DS_ = 0 V) as well as a photoillumination (white light with a photointensity of 4000 Lux). It can be seen that all devices show comparable and excellent stability under NBIS with |ΔV_TH_| < 1 V.

## 4. Conclusions

In conclusion, ITO TFTs with Al, ITO, Ti, and Mo source/drain electrodes were fabricated in this work. The results show that the ITO TFT with an Al electrode exhibits better device performance than the other devices. The major electrical properties of the device with an Al source/drain electrode are a µ_FE_ of 26.45 cm^2^/Vs, a V_ON_ of 2.7 V, and a SS of 201.50 mV/decade. However, the devices with the four electrode materials all reveal excellent stability under NBIS with |ΔV_TH_| < 1 V. All the results indicate that ITO TFTs with an Al source/drain electrode have great potential in future display applications.

## Figures and Tables

**Table 1:** Electrical parameters of ITO TFTs, including µ_FE_, SS, and V_ON_.

Source/Drain	µ_FE_ (cm^2^/Vs)	SS (mV/Decade)	V_ON_ (V)
Al	26.45	201.50	2.7
ITO	20.83	108.70	3.9
Ti	12.46	179.00	4.5
Mo	7.99	113.39	5.3

**Table 2:** Summary of recent reports for major electrical parameters of oxide TFTs with different source/drain electrodes.

Source/Drain	Channel	R_SD_ × W (kΩ·μm)	µ_FE_ (cm^2^/Vs)	SS (mV/Decade)	V_ON_ (V)
Al [35]	IGZO	>500	8.1	1000.09	2.5
ITO [25]	IGZO	704	9.1	704	~0
ITO [36]	STO	/	5.6	300	−2.4
Cu/Ti [37]	IGZO	3200	18.8	/	~2
Cu [34]	IGZO	120	6.3	/	~−3
Cu/ITO [38]	IGZO	18	11.5	200	1.5
Mo [39]	IZTO	/	35.25	210	−0.58
Au [40]	IAZO	/	20.57	550	~0
CuCrZr [41]	NdIZO	84.5	32.1	160	~0
Al [this work]	ITO	109.4	26.45	201.50	2.7

## Data Availability

The data that support the findings of this study are available from the corresponding authors upon reasonable request.
